# Using mouse models to study function of transcriptional factors in T cell development

**DOI:** 10.1186/2045-9769-1-8

**Published:** 2012-10-10

**Authors:** Peng Li, Yiren Xiao, Zhixin Liu, Pentao Liu

**Affiliations:** 1_8 Key Laboratory of Regenerative Biology, Guangzchou Institutes of Biomedicine and Health, Chinese Academy of Sciences, Guangzhou, China; 2_8 Guangdong Provincial Key Laboratory of Stem Cell and Regenerative Medicine, Guangzhou, China; 3_8 Wellcome Trust Sanger Institute, Hinxton, Cambridge, CB10 1HH UK

**Keywords:** Genetic modified mice, Humanized mice, Lymphopoiesis, T cell development, Bcl11b

## Abstract

Laboratory mice have widely been used as tools for basic biological research and models for studying human diseases. With the advances of genetic engineering and conditional knockout (CKO) mice, we now understand hematopoiesis is a dynamic stepwise process starting from hematopoietic stem cells (HSCs) which are responsible for replenishing all blood cells. Transcriptional factors play important role in hematopoiesis. In this review we compile several studies on using genetic modified mice and humanized mice to study function of transcriptional factors in lymphopoiesis, including T lymphocyte and Natural killer (NK) cell development. Finally, we focused on the key transcriptional factor Bcl11b and its function in regulating T cell specification and commitment.

## Introduction

Lab mice are invaluable tools in modern biomedical research because of its short generation time, small size and prolificacy in breeding. A pure genetic background of the inbred mouse strain greatly improves the reproducibility of experiments, as individuals from the same mouse strain are genetically identical. Currently, all the common inbred strains in labs have been inbred for at least 80 generations since their original isolation; thus the genomes of all siblings of the same inbred line are essentially identical [1]. These inbred mouse strains provide good platforms for studying the immune system and modeling human immune disease [2–4]. In thymus, hematopoietic cells undergo several developmental processes to give rise to variety of T subsets which is one of the central players in orchestrating immune responses. Rothenberg and Taku Naito have reviewed a combination of transcription factors, including *E2A, GATA3* and *TCF1*, which are essential to initiate T cell differentiation program [5]. However the key factor of T lineage specification and commitment in vivo is to be understood. Recently, Bcl11b, a transcriptional factor, was identified to be essential for T cell development and for the maintenance of T cell identity [6–8]. Deletion of Bcl11b in T cells can cause these cells to reprogramme into NK cells in mice [6]. Our goal here is to give an introduction on the function of transcriptional factors, especially Bcl11b, in T cell development

### Genetic manipulation of the mouse genome

#### Over-expression of targeted genes—gain-of-function

A most breakthrough advantage of using the mouse to study the immune system and to model human disease is the availability of a range of genetic technologies. In 1981, several groups produced transgenic mice by injecting transgenic DNA into mouse pronuclei [9, 10]. Importantly, DNA introduced into the mouse genome by this method results in establishment of the transgene in the germ line. This technology offers scientists the opportunities to perform gain-of-function studies for specific genes in the mouse model. Currently, DNA fragments can be conveniently inserted into the host cell genome by virus [11] or transposon system, such as Sleeping Beauty [12] and PiggyBac [13, 14]. Woltien K and his colleagues successfully reprogrammed murine and human embryonic fibroblasts to induced pluripotent stem cell (iPS) using doxycycline-inducible transcription factors delivered by PB transposition [15]. However, the copy number and the genomic position of the transgene that integrates into the mouse genome vary in different operation. Therefore, it is difficult to control the expression levels or patterns of the transgene [16]. In addition, except bacterial artificial chromosome transgenes [17], transgenes usually are not large enough to contain all of the cis-acting elements for recapitulating the endogenous gene expression patterns [18]. In a word, loss-of-function assays are usually recruited to study gene functions besides gain-of-function assays.

### Silent target genes—loss-of-function

**Figure 1:**
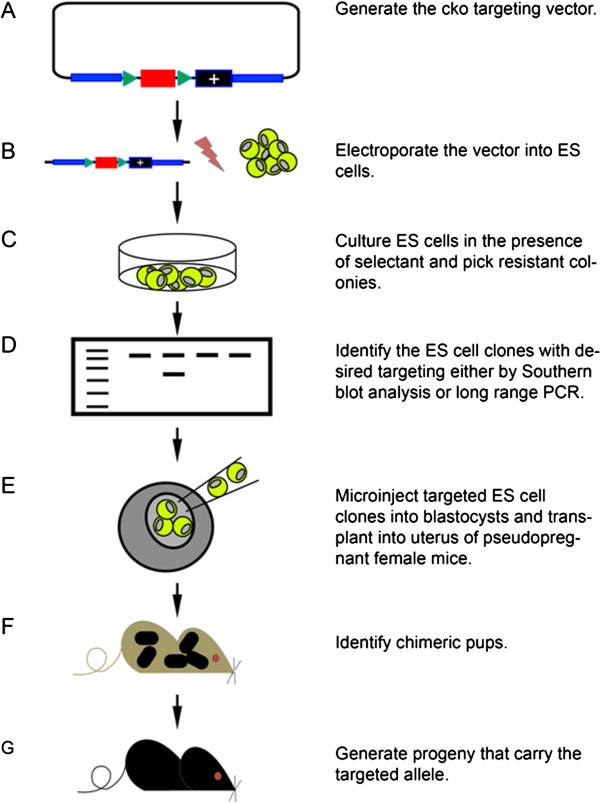
**General procedure for generation of a conditional knockout mouse strain by gene targeting strategies.** (**A**) Generation of a targeting vector containing critical exon of targeted genes (red rectangle), two loxP site (green triangle), a positive (+) selection cassette and sequences of homology with the target locus (blue line). (**B**) The vector is linearized and electroporated into ES cells. (**C**) Correct tranformants are selected for in the presence of a selectant (eg. G418 if a neomycin resistance cassette is present in the targeting vector). (**D**) Correctly targeted ES cell clones are then identified and genetically characterized using long range PCR or Southern blot analysis. (**E**) The selected ES cell clones are then microinjected into 3.5 dpc blastocysts and transplanted into the uteri of pseudopregnant females. (**F**) Chimeras obtained from the microinjections are mated with wild-type mice to establish germ-line transmission of the modified allele. (**G**) Progeny derived from the chimeras are characterized using long range PCR or Southern blot analysis, and a mutant mouse line that carries the desired targeted allele is established.

In 1981, pluripotent mouse embryonic stem cells (ES) were isolated from the inner cell mass of 3.5 days post-coitum (dpc) wild type mouse embryos [19, 20]. Later, it was demonstrated that these ES cells were able to contribute to the germ line in chimera mice derived from ES cells, even after genome modification by retrovirus [21, 22]. Precise manipulation of the mouse genome was achieved by demonstrating that homologous recombination works efficiently in mouse ES cells [23, 24]. Thus a combination of mouse ES cell manipulation and homologous recombination technologies gave birth to ‘gene targeting’ which has revolutionized mouse genetics. With birth of genetically engineered ‘knockout’ mice, target mutations have been introduced to many loci to study gene functions in the immunity. In 1990, Smithies’ and Jaenisch’s group independently generated β2-microglobulin knockout mice in which expression of major histocompatibility complex (MHC) class I molecules was abolished. The mutant mice developed normally but had severely reduced number of CD8^+^ cytotoxic T lymphocytes (CTLs), indicating that MHC class I molecules are required for the selection of MHC class-I-restricted CD8^+^ T cells and for antigen recognition by these cells, but not necessary for T cell development in general [25, 26]. It is now feasible to knockout every single gene in the mouse and observe phenotype in this knockout mice (Figure 1) [27].

Conventionally, homologous recombination is employed in gene targeting to insert a designed mutation into the homologous recombination loci in mouse ES cells. These ES cells are totipotent and when injected into mouse blastocytes, these cells can differentiate into all cell types of a chimeric mouse [28]. In addition, homologues knockout of some important gene, such as the house keeping gene, leads to embryonic death [29], exposing the defect of knockout technology, as we lost the opportunist to study function of these genes in adult mice. Conditional knockout mice approaches, often using the Cre-loxP site-specific recombination system, were developed to overcome the embryonic lethality problem and to investigate gene function temporally and spatially [30, 31]. For example, T cell development is arrested at early stages in *Notch1* and *Gata3* adult CKO mice, demonstrating the importance of these two genes for T cell development [32]. However, either *Notch1* or *Gata3* deleted mice are died during embryonic period [33, 34]. Similarly, conditional knock out of Bcl11b in double negative (DN) 3 T lymphocytes induce DN3 T cell differentiate to NK cell [6], but Bcl11b in conventional knockout mice die within the first day after birth [35].

### Humanized mice

Although the mouse is instrumental in elucidating key processes in the immune system and revealing molecular mechanisms of immune diseases [36, 37], ethical issues and significant species differences between mouse and human constrain researchers to study human immune system in vivo. Humanized mice, or mouse-human chimaeras, have been developed to overcome these constraints and are now an important research tool for the in vivo study of human hematopoietic system. Humanized mice are classified into two kinds: one is immune deficient mice engrafted with hematopoietic cells or tissues, that the other is transgenically expressing human genes. And the latter have been previously reviewed [38]. In this review, humanized mice refer to immune deficient mice that are engrafted with hematopoietic cells or tissues. Many important research advances have been made using humanized mice as models to study human hematopoietic system. For example, Faiyaz Notta and his colleagues purified so far the most accurate human HSC Lin^−^CD34^+^C D38^−^CD45RA^−^Thy1^+^Rho^lo^CD49f^+^ at single cell resolution in immune deficient mice [39].

Several mouse strains have so far been used as recipients of human hematopoietic cells. *Scid* (severe combined immune deficiency) mice carry a spontaneous loss of function mutation of the *Prkcd* ^*scid*^ gene, or *scid* mutation which results in severely deficient in T and B lymphocytes and aberrant adaptive immune system [40]. Consequently, *scid* mice can engraft human hematopoietic stem cells/progenitors, mature lymphocytes and tumor cells without rejection [41]. Though *scid* mice were proved to be successful, the engraftment efficiency was usually low due to high levels of innate immunity response in mice [42]. To overcome this problem, Shultz LD and his colleagues [43] generated the NOD-*Scid* mice (NOD/LtSz*PrKdc* ^*scid*^ */PrKd* ^*scid*^) strain by introducing the *scid* mutation from CB17-*Scid* mice into the NOD strain mice that contain reduced NK cell activity [44]. However, NOD-Scid mice are still not good enough in xenograft for the residual innate immunity [43]. The interleukin-2 receptor γ-chain (Il2rg) locus is an essential component of the receptor for IL-2, IL-4, IL-7, IL-9, IL-15 and IL-21 [45]. Loss-of-function mutation at the IL-2R γ-chain results in severe impairments in T- and B-cell development and function, and completely prevents NK-cell development [46]. Two sub-strains—NOD/ShiLtSz-*scid*/*IL2R*γ^*null*^ (-NSG) and NOD/ShiJic-*scid*/*IL2R*γ^*null*^ (NOG) which can engraft higher human grafts were generated by introducing Il2rg-null mutations to NOD- *Scid* mouse independently. [47, 48]. Both NSG and NOG strains are widely used for studying human hematopoiesis and leukemiagenesis [49].

### Hematopoiesis

Hematopoiesis is the dynamic and complex developmental process of the formation of new blood cells, including red blood cells (erythrocytes), white blood cells (leukocytes), and platelets [50]. For mammalians, hematopoiesis can be divided into two periods, the primitive hematopoiesis and definitive hematopoiesis. Primitive hematopoiesis is referred as hematopoiesis taking place prior to the development of the fetal liver. The term definitive hematopoiesis is used to describe blood formation after the formation of the fetal liver. In the mouse embryos, primitive hematopoiesis formed at 8.0 days postconception (dpc 8) in the yolk sac blood islands. During embryonic development, the location of hematopoiesis shifts from the aorta-gonad-mesonephros (AGM) region to the fetal liver [51]. After birth, the hematopoiesis center migrates from the fetal liver to the bone marrow (BM), where the special microenvironment (the endosteal niche) supports hematopoiesis [52].

**Figure 2:**
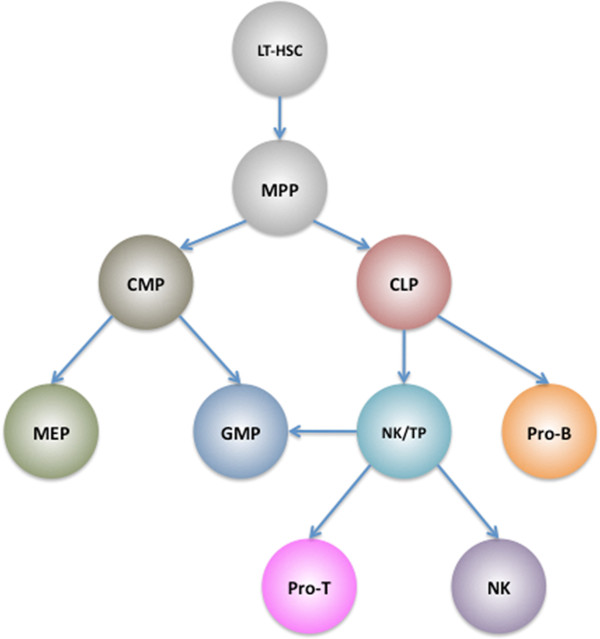
**Current scheme of haematopoiesis.** LT-HSC, long-term hematopoietic stem cell; MPP. Multipotent progenitor; CMP, common myeloid progenitor; CLP, common lymphoid progenitor; MEP, megakaryocyte erythroid progenitor; GMP, granulocyte macrophage progenitor; NK/TP, NK/T progenitor, which have NK, T, and myeloid cell potentials. Pro-T and Pro-B are progenitor cells that go through several stages to eventually produce T and B cells. Arrows indicate cell differentiation.

HSCs are a heterogeneous pluripotent population and are composed of long-term HSC and short-term HSC. Long-term HSCs are capable of self-renew for the whole life of the host, whereas short-term HSCs retain self-renewal capacity for approximately 8 weeks [53]. Short-term HSCs proliferate and differentiate to multipotent progenitors (MPP) that give rise to common myeloid progenitors (CMP) and common lymphoid progenitors (CLP). CMP give rise to granulocyte macrophage progenitors (GMP) and megakaryocyte erythroid progenitors (MEPs) [54]. GMP can differentiate towards neutrophils, monocytes, macrophages, eosinophils, basophils, and mast cells, while MEP generate megakaryocytes and erythrocytes [55]. In contrast, CLP are restricted to give rise to T cells, B cells, Natural Killer (NK) cells, and some dendritic cells, but two studies suggest that some progenitors derived from CLP retain myeloid differentiation potentials (Figure 2) [56, 57]. However, cell lineage can be studied by over-expression or inactivation of some key transcriptional factors in hematopoietic lineages [58]. Ectopic expression of Gata1 forced primary myeloid progenitors differentiate to erythroid, eosinophil, and basophil-like cell lineages, instead of macrophages and granulocytes [59]. Similarly, expression of PU.1 and C/EBPα in early mouse T cells endows the lymphoid T cell with myeloid differentiation potential [56]. B and T lymphocytes can be reprogrammed to myeloid cells upon over expressing PU.1, C/EBPα and/or C/EBPβ [60, 61].

### T cell development

T cells are derived from the thymus and are important for adaptive immune responses [62]. T cells are capable of recognizing antigen peptides that are bound to MHC molecules. There are two main subpopulations of T cells: CD4^+^ helper T and CD8^+^ cytotoxic T cells. CD4^+^ T cells recognize MHC II molecule complexes and secrete various cytokines after being activated. Conversely, CD8^+^ T cells recognize MHC class I molecules and exhibit cell-killing activity after activation [63, 64]. Acquired Immune Deficiency Syndrome (AIDS) provides a vivid and tragic illustration of the importance of T cells in immunity. The human immunodeficiency virus (HIV), the causative agent of AIDS, binds to the CD4 molecules which causes depletion of CD4^+^ T cells in AIDS patients [65]. Without CD4^+^ T cells, AIDS patients become hypersusceptible to pathogens that normally inhabit in tissues without much harming, and die of the opportunistic infections [66].

**Figure 3:**
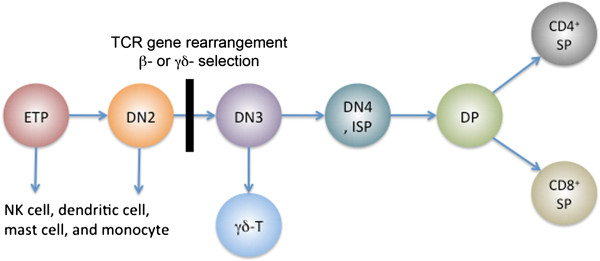
**Stages in T cell development.** Early T cell precursors (ETPs) differentiate from double negative (DN) to double positive (DP) to single positive (SP) stages. Arrows indicate cell differentiation. Note that ETP and DN2 thymocytes contain non-T-cell options. **β**- and **γδ**- selection occurs during the accumulation of the DN3 T cells.

T cell development, which happens in the thymus, involves progenitor homing, lineage specification and commitment [67]. It also requires the intrathymic microenvironment and interactions among key transcription factors [5, 68]. In adult mice, CLPs migrate from the bone marrow to the thymus and initiate the program of T cell development [69]. In the thymus, thymocytes are classified into three distinct maturational---double negative (DN; CD4^-^CD8^-^), double positive (DP; CD4^+^CD8^+^) and single-positive (SP; CD4^-^CD8^+^ or CD4^+^CD8^-^) (Figure 3) [70].

Classically, the DN population can be further subdivided into four subtypes (DN1—4) based on the level of markers CD117 (c-Kit), CD44, and CD25 expressed on the surface of the cell [71]. The CD117^+^DN1 (CD44^+^CD25^-^) subsets, also known as early T cell precursors (ETP), are thought to contain not only T lineage potential but also B lineage and granulocyte-monocyte potential [56, 72]. Flt3/Flk2, a tyrosine kinase receptor, endows ETP the choice to differentiate into not only T cell, but also B cell, NK cell and macrophages [73]. In addition, both Gata3 (GATA-binding protein 3) and Bcl11b are essential for ETP maintenance and development [7, 74]. Notch signaling is indispensable for ETP [75]. Recently, T-cell factor 1 (TCF-1; also known as transcription factor 7, T-cell specific, TCF7) was identified as a messenger that transmit the commands from Notch signal to downstream transcriptional factor like Gata3 and Bcl11b during T cell specification and differentiation [76].

T cell specification occurred in the course of transition from ETP to DN2 (CD44^+^CD25^+^). During this period, several key T cell genes, such as *Bcl11b*, *Tcf12* (*HEBAlt*), *Gata3* and *Notch1* are up-regulated. DN2 T cells notwithstanding, retain the potentials to differentiate into NK cells and myeloid cells [56, 57, 77]. ETPs and DN2 thymocytes initiate TCR gene rearrangements. However, they do not exhibit full V(D)J rearrangements or express any TCRβ or TCR γδon their cell surface, which are symbols of committed T cells. The non-T cell potentials are lost in the DN3 (CD44^-^CD25^+^) T cell stage [73]. Some DN3 T cells successfully rearrange TCR γ- and δ-chains instead of β-chain and differentiate into γδ-T cells. The majority of T cells at DN3 stage commit to the αβ-T cell lineage by further upregulating key T cell genes and shutting down expression of genes that are important for non-T cell lineages [78]. Co-activities of E2A and HEB are not only crucial for TCR rearrangement [79], but are also essential for T cells to regulate β selection, positive selection and lineage-specific gene expression at the subsequent T-cell receptor checkpoint [80]. The DN4 (CD44^-^CD25^-^) thymocytes undergo β-selection after successful TCR βgene rearrangements and initiate the differentiation to CD4^+^CD8^+^ double positive (DP) cells [81].

Positive selection of the developing T-cell receptor repertoire occurs in the thymic cortex where DP cells (CD4^+^CD8^+^) selected via MHC class II and MHC class I molecules will eventually become CD4^+^ cells and CD8^+^ cells, respectively [82]. During negative selection, that strong interaction between thymocytes and antigens would trigger Fas apoptotic signal, which in turn encourage apoptosis of the thymocytes [83]. After positive and negative selections, the surviving thymocytes migrate to the peripheral lymphoid tissues where cytokines like IL-7 and the constant interaction between T cell and self-peptide MHC play a critical role in T cell maintenance [84]. Moreover, thymic epithelial-cell microenvironments are indispensible for T cells selection and maintenance during immune responses [85]. Some transcriptional factors are required in T cell maturation. For example, E protein transcription factors HEB and E2A are vital in DP to SP transition [86]. Besides the transcriptional factors, establishment of T cell identity is also regulated by epigenetic modification. For instance, PU.1 and GATA3 that are essential for T cell development are controlled by dynamic histone modification at promoter region [68].

### NK cell development

NK cell were first discovered by Rolf Kiessling in the 1970s, when he found a small population of large granular lymphocytes displayed cytotoxic activity against a wide range of tumor cells in the absence of any previous immunization with the tumors [87, 88]. This population of cells were named "natural killer" because of the initial notion that they did not require prior activation to kill cells. It was later shown that NK cells preferably targeted cells that expressed low levels of MHC class I molecules, a concept termed as "missing-self" recognition [89, 90]. NK cells constitute an essential component of the innate immune system, which, unlike the adaptive immune system, recognizes and responds to pathogens without requiring prior priming through clonal antigen receptors. Mice with deficiencies in NK stimulatory immune-receptors such as NKG2D and DNAM-1 are defective in tumor surveillance in models of spontaneous malignancy [91].

NK cell development occurs mainly in BM, although a bipotent T/NK progenitor (TNKPs) containing T- and NK-cell potentials has been identified in mouse fetal liver and fetal thymus [92]. In BM, CLP commit to natural killer cell precursors (NKPs), which are defined as Lin^-^(lineage)CD122^+^NK1.1^-^DX5^-^[93]. Upon CD122 expression, NKPs lose B-, T- or myeloid-cell potentials and respond to IL-15 stimulation, which promotes NK cell development [94]. Then, NKPs further differentiate into immature NK cells that express NK1.1 in bone marrow and liver [95, 96]. Finally, immature NK cells differentiate into mature NK cells and obtain NK-cell self-tolerance by expressing inhibitory NK-cell receptors like Ly49 and CD94-NKG2. [97]. Mature NK cells circulate in the body as defenders to maintain homeostasis and shape their weapons by producing Fasl, Trail, γInterferon- IFN-γ perforin and granzymes [98]. Thymic NK cells express both GATA3 and CD127 (IL-7Rα). Compared to conventional NK cells, the cytotoxicity in thymic NK cells is compromised [99]. Moreover, it was reported that NK cell progenitors (CD34^+^CD1a^−^) in human thymus that eventually differentiated into functional CD56^+^ NK cells express bone morphogenetic protein (BMP) receptors IA (BMPRIA), suggesting that BMP signaling pathway was required for human thymic NK development [100]. In addition, NKp46, a NK cell marker encoded by *Ncr1* gene, was found capable of blockading enhanced NK cell reactivity [101]. Recent studies proposed that Bcl11b was a key regulator in controlling NK cell development as loss of Bcl11b induced T cell reprogrammed to NK like cell [6, 102].

### Bcl11b

**Figure 4:**
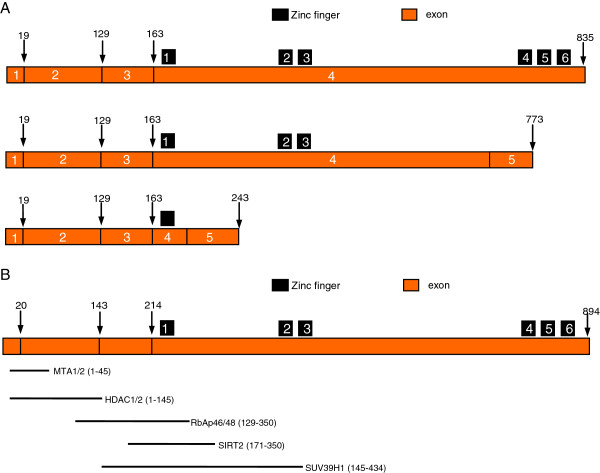
**Structure of human Bcl11b protein.** **A**: The bar represents the 3 isoforms of Bcl11a. Exons 1-3 are the same in all the 3 isoforms. Isoform 1 is the longest one containing 835 amino acids. The 4th exon contains all the 6 Zinc-fingers domains. The longest isoform comprised exons 1-4, whereas the 2 common shorter isoforms exhibited alternative splicing from within exon 4 to an additional exon 5. **B**: The bar represents the isoform 1 of Bcl11b including 4 exons and 6 Zinc-finger domains. All the 6 Zinc-finger domains are distributed within the longest exon 4, and the exons 1–3 are in length of 214 amino acids. The 2^nd^ and 3^rd^ Zinc-finger domains are responsible for DNA binding. The regions shown by lines encodes domains that bind to various Bcl11b cooperating proteins, such as HDAC1/2 (histone deacetylase), SIRT2 (sirtuin family protein), SUV39H1 (histone methyltransferase), MTA1/2 (metastasis-associated proteins), RbAp46/48 (Rb-associated protein).

The B-cell lymphoma/leukemia 11 (Bcl11) family has two members, Bcl11a and Bcl11b. Both of them are Kruppel-like C2H2 type zinc finger transcription factors [103]. Bcl11a was first discovered as a retroviral insertion site (*Evi9*) in myeloid leukemia tumors in the BXH-2 mouse [104]. Located at 2p16.1, *Bcl11a* is consists of 5 exons and encodes three different isoforms (Figure 4A). The first three exons of *Bcl11a* present in all the three isoforms. The longest isoform comprised exon 1-4, whereas the rest two common shorter isoforms exhibited alternative splicing from within exon 4 to an additional exon 5. However, the exon 5 in the isoform 2 is different from the one in the isoform 3 due to frame shift in their exon 4.

Bcl11a is also expressed in early T cell progenitors and is also important for T cell development. Germ-line deletion of *Bcl11a* causes neonatal lethality and an absence of B cells at the earliest B cell development stages [105, 106]. Recipient mice of *Bcl11a*-deficient fetal liver cells develop T cell leukemia [105]. Genome-wide association studies in human have revealed association of the *Bcl11a* locus with persistent fetal hemoglobin in the adult [107, 108]. Further characterization of Bcl11a mutant mice also uncovers the key role of Bcl11a in the fetal to adult expression switch of hemoglobin [109]. Beside of its important functions in B cell development and the switch of hemoglobin expression, Bcl11a is related to pancreatic beta-cell function, dysfunction of which leads to type 2 diabetes [110]. In addition, a recent study shows that Bcl11a is required in neuronal morphogenesis and sensory wiring of the dorsal spinal cord [111].

*Bcl11b*, also named *Rit1* (radiation-induced tumor suppressor gene 1), is the other member of the *Bcl11* family in the mouse and human genomes. *Bcl11b* located at 14q32.2, is consists of 4 exons and encodes 2 isoforms. Compared to the isoform 1 of BCL11B that contains 894 amino acids, its isoform 2 lacks exon 3 and only have 823 amino acids. All the 6 zinc fingers are distributed within the exon 4. Alejandro Gutierrez and his colleagues find that only exon 2 and 3 are responsible for encoding the DNA binding domains [112]. some other domains within the Bcl11b participate in protein-to-protein interactions, including histone deacetylase (HDAC1 and HDAC2) [113], sirtuin family protein (SIRT2) [114], metastasis-associated proteins (MTA1 and MTA2) [115], Rb-associated protein (RbAp46 and RbAp48) [116] and histone methyltransferase (SUV39H1) [113] (Figure 4B).

*Bcl11b* was initially identified as a tumour suppressor gene in T cells, because loss of heterogynous (LOH) contributed to the formation of thymic lymphomas in γ–ray irradiated mice [117, 118]. In addition, BCL11B is found to be involved in human T cell leukemia. A specific cryptic translocation, t(5;14)(q35.1;32.2), accrued in about one fifth of T-cell adult leukemia/lymphoma (T-ALL) patients, serves to activate expression of *Hox11L2* by juxtaposition with strong T cell enhancer elements at the 3’ of the *Bcl11b* locus [119, 120]. Further more, a novel chromosomal aberration, inv (14) (q11.2q32.31) was reported in T-ALL samples. In this inversion, the 5’ part of *Bcl11b*, including exons 1-3, was joined to the TRDD3 segment of the TCR δlocus. Consequently, in-frame transcripts with truncated human BCL11B and TCRδconstant region were highly expressed in screened T-ALLs but not in normal T cells [121]. Interestingly, though Bcl11b *i*s considered as a tumour suppressor gene, it is highly expressed in many human T cell tumour lines and is required for their survival. Suppression of Bcl11b by RNA interference (RNAi) causes apoptosis of these tumour cells, possibly due to a decrease of a cell-cycle inhibitor, p27, and an anti-apoptotic protein, BCL-xL. This indicates involvement of the mitochondrial apoptotic pathway. In contrast, normal mature T cells remained unaffected within the experimental time period [122, 123]. Therefore, Bcl11b could be an attractive therapeutic RNAi target in T-cell malignancies [124].

### Function of Bcl11b in early T cells

During evolution, a homolog of Bcl11b first appeared in cartilaginous fishes. In sea lampreys, a jawless vertebrate, expression of a Bcl11b ortholog is specifically detected in VLRA^+^ cells that are similar to T lymphocytes in vertebrates, but not in VLRB^+^ cells that are similar to B lymphocytes in vertebrates [125]. In bony fish, the Bcl11b ortholog is expressed in the thymus and positively regulates *Ccr9* expression, which encodes the receptor for ccl25, a novel chemokine expressed in thymic epithelium [126]. In both the mouse and human, Bcl11b is highly expressed in T cells [35, 119]. In the hematopoietic system, Bcl11b was absent in B cells, myeloid cells and most NK cells, but highly expressed in T cell lineage [7]. Developmentally, its expression is tightly associated with T cell commitment. Early thymocytes, for example, start to express Bcl11b during the transition from ETP to DN2b stage, at a time when early thymocytes gradually lose non-T-cell potentials, by histone 3 methylation (H3) and demathylation [68], implicating that Bcl11b plays an important role in this process. Bcl11b expression is maintained at high levels in T cells beyond DN2b stages [106]. In mice, over-expression of Bcl11b results in the differentiation of T-helper (Th) cells [127], whereas down-regulating or knockout of *Bcl11b* induces reprogram from T cell into NK-like cell [6]. However, function of Bcl11b in human T cell requires further investigations.

Loss-of-function studies on Bcl11b in the mouse demonstrated that Bcl11b is required for early T cell development and the survival of T cells. Bcl11b homozygous mutant knockout mice die in the first few days after birth possibly due to neurological or other uncharacterized defects [35, 128, 129]. Furthermore, in this *Bcl11b*-deficient strain, T cell development is blocked at the DN2-DN3 stage without obvious defects in other hematopoietic lineages. A recent study using the same knockout strain shows that *Bcl11b*-deficient thymocytes are arrested at DN2 stage and acquires self-renewal properties in vitro [8]. Our results, together with a study from Rothenberg’s lab, show that Bcl11b-deficient DN1 and DN2 thymocytes stop T cell development and acquire NK differentiation potentials in mouse thymus. We thus named these killer cells that were reprogrammed from T cells as induced T-to–natural killer (ITNK) cells [6]. In *Bcl11b*-deficient thymocytes show impaired Vβ to Dβ rearrangements and absence expression of pre-T cell receptors (pre-TCR) complex on the cell surface [35]. In addition, thymocytes undergo profound apoptosis in neonatal *Bcl11b*-deficient mice [35]. However, apoptosis is unlikely to be the main reason for the failure of T cell development upon loss of Bcl11b, because inactivation of p53 in mutant thymocytes is not sufficient to fully restore the T cell development, even though some immature single-positive (ISP) T cells are indeed detected in *Bcl11b* ^*-/-*^ *p53* ^*-/-*^ embryos [130]. The exact cause of T cell defects in *Bcl11b*-deficient mice thus remained unresolved. Taken together, Bcl11b is required for the early T cells to sustain T cell development and suppressed non-T cell differentiation potentials.

### Function of Bcl11b in mature T cells

Bcl11b plays a critical role in DP thymocytes by controlling positive selection of both CD4 and CD8 lineages. *Bcl11b*-deficient DP thymocytes are prone to spontaneous apoptosis, which is possibly due to impaired proximal TCR signaling and attenuated extracellular signal-regulated kinase phosphorylation and calcium flux that are required for initiation of positive selection [131]. In mice CD4^+^ T cells, following activation through TCR, down-regulation of endogenous human BCL11B reduces IL-2 expression, whereas overexpression of BCL11B augments IL-2 expression [132]. Further studies indicate BCL11B promotes IL2 expression by binding and activating the IL2 promoter through the US1 site and by enhancing NFKB1 (NK-kappa-B) activity [133]. For the CD8^+^ T cells, Bcl11b is essential in the antigen-specific clonal expansion and cytolytic effector function [134]. Loss of Bcl11b can also induce mature T cells, like DP and CD8^+^, to differentiate into ITNK [6]. It has been suggested that Bcl11b represses gene expression program that are associated with mature CD4^+^ and CD8^+^ thymocytes, including Zbtb7b (Th-POK) and Runx3 that are important for the development of mature CD4^+^ and CD8^+^ T cells, respectively [135–137].

It is worth mention that Bcl11b haploinsufficiency is demonstrated in thymocyte development. Compared to wild type mice, heterozygous Bcl11b mutant mice have fewer thymocytes. These *Bcl11b* ^*+/-*^ thymocytes are much more prone to lymphomagenesis [138]. Moreover, in γ-irradiated mice, *Bcl11b* heterozygosity promotes clonal expansion and differentiation arrest of thymocytes [139]. Nevertheless, it was puzzling that no tumour development has ever been reported in mice transplanted with Bcl11b homozygous knockout progenitors from fetal live.

### Reprogramming from T cells to NK-like cells

**Figure 5:**
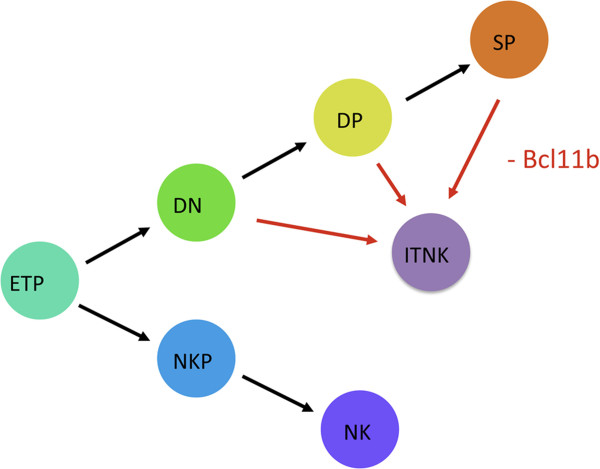
**Summary of reprogram from various T cell subsets to ITNK cells.** ETP, early T cell precursors; DN, double negative; DP, double positive; SP, single positive; ITNK, Induced T to natural killer cells; NKP, natural killer progenitors; NK, natural killer cells. Black arrows indicate differentiation and red arrows indicate reprogramming.

Using Bcl11b conditional knockout mice, it was demonstrated that Bcl11b is important for both early and mature T cell development. Positively regulated by Notch signaling, Bcl11b suppresses key NK genes expression and promotes the expression of cocktail of T cell-associated transcription factors in T cells. Thus, acute loss of Bcl11b makes early T cells lose their T cell potential and differentiate to NK cells. In committed DN3 thymocytes, Bcl11b is essential for the maintenance of T cell identity, since every single survival Bcl11b deficient DN3 T cell abandons its T cell development program and subsequently reprograms to ITNK. Similarly, mature T cells transdifferentiate to ITNK upon loss of Bcl11b. In a word, Bcl11b is indispensable for T cells at all developmental stages to sustain T cell development and maintain T cell identity, while suppress NK cell program (Figure 5) [6].

### Bcl11b in other tissues

Besides the immune system, Bcl11b is also required in skin, neuron and tooth development [18, 129, 140]. Bcl11b is highly expressed in mouse skin during embryogenesis. In the developing epidermis at late stage of fetal development and in the adult skin, Bcl11b expression decreases and becomes restricted to the proliferating cells of the basal cell layer [17, 128, 129, 140]. Further analysis indicates that a subset of skin stem cells may express Bcl11b [140, 141]. Analysis of mice with gremline deletion of *Bcl11b* shows that Bcl11b is required in skin during development, particularly in keratinocyte proliferation and late differentiation events [140–142]. Similarly, BCL11B is expressed in human epidermis, and is linked to disease progression and/or maintenance in atopic dermatitis and allergic contact dermatitis patients [143].

In the nerve system, Bcl11b is essential for the development of corticospinal motor neurons axonal projections to the spinal cord in vivo [128], and is also indispensable for striatal medium spiny neurons differentiation, striatal patch development, and the establishment of the cellular architecture of the striatum [129]. In humans, BCL11B expression is maintained at high levels in normal adult striatum but significantly decreased in Huntington disease (HD) cells, because mutant Huntington striatal neurons is sensitive to over-expression of BCL11B [144], suggesting that sequestration and/or decreased BCL11B expression is responsible for the deregulation of striatal gene expression and the specificity of pathology that are observed in HD.

Bcl11b also participates in the regulation of epithelial cell differentiation during tooth morphogenesis and is highly expressed in ectodermic components of the developing tooth. *Bcl11b*-deficient mice show multiple defects at the bell stage and have abnormal incisors and molars [142]. In addition Bcl11b plays multiple roles in modulating cell migration, cell proliferation and hair follicle stem cell maintenance during cutaneous wounding [145].

### Partners and downstream genes of Bcl11b

Bcl11b, also termed Ctip2 (COUP-TF interacting protein), was independently isolated for its ability to interact with all members of the chicken ovalbumin upstream promoter transcription factor (COUP-TF) subfamily of orphan nuclear receptors [146]. COUP-TFs usually mediate transcriptional repression by recruiting nuclear receptor co-repressor (NCoR) and/or silencing mediator for retinoid and thyroid hormone receptor (SMRT) to the template. COUP-TF family members play important roles in pattern formation in the developing nervous systems of xenopus and drosophila [146–148]. As a COUP-TF-interacting protein, Bcl11b mediates transcriptional repression when tethered to a promoter by interacting with a DNA binding protein, such as ARP1, which is a member of COUP-TF subfamily of orphan nuclear receptors [146]. BCL11B also interacts with two metastasis associated proteins MTA1 and MTA2 directly [115]. In HEK293 cells, BCL11B recruits sirtuin 1 (SIRT1) that is a trichostatin-insensitive, nicotinamide-sensitive class III histone deacetylase to the promoter region of a reporter gene template [114, 149]. In addition, BCL11B recruits histone deacetylase 1 (HDAC1) and HDAC2 to promote local histone H3 deacetylation at the HIV-1 promoter region [113].

To regulates cell cycle. Bcl11b cooperates with SUV39H1 and histone methylase to silence *Cdkn1a* (*p21*) [150]. *Cdkn1a* (*p21*) is a major cell cycle regulator which can respond to DNA damage senescence and tumor suppression [151]. In addition, Bcl11b can also repress another cyclin-dependent kinase inhibitor, Cdkn1c (p57KIP2) [152], which is able to associate with and inhibit the catalytic activity of a number of cylin-cdk complexes [153]. A recent study proposes that the up-stream regulator of Bcl11b is TCF-1 which is activated by Notch1 signals [76].

## Conclusions

The Immune system is the central defender against outside pathogen and neoplasm. The importance of human immune system cannot be too much investigated. So far, millions of patients have been saved for the newly found drugs which are screened or designed by the mounting information about immunological response and tumourgenesis. However, progresses are impeded by in vivo study of human immunology and ethic constrains. Since the genome and their the immune system between human and mouse is similar, making mouse the most widely used model in elucidating key processes of the immune system and in revealing molecular mechanisms of immunological diseases [36, 37]. Based on gene manipulation and humanize mice, the mask of many genes related to immune and hematopoietic system, including *Bcl11b, Gata3* and *TCF1,* are unveiled. So far, programs of the whole genome knockout mice, like the Knockout Mouse Project (KOMP) and the European Conditional Mouse Mutagenesis Program (EUCOMM), have generated thousands of conventional and conditional knockout mouse strains and are distributing them worldwide [154]. Furthermore, with the development of zinc nuclease fingers and TALENs technologies, it is easier to generate gene knockout and knock-in mice. Thus, we expect, in the coming years, the genome engineering technologies and conditional knockout mouse strains will be available for more labs and researchers, so that functions of more transcription factors will be elucidated in T cell development.
